# Shifting the focus from animal species to livestock production systems: an interactive tool for evaluating food contributions relative to environmental impacts

**DOI:** 10.1093/af/vfaf008

**Published:** 2025-04-05

**Authors:** Elna de Lange, Lindeque du Toit, Andrew Fletcher, Taras Iliushyk, Bohdana Kalinovska, Naomi Lupton, Enrike Maree, Peer Ederer

**Affiliations:** GOALSciences – Global Observatory on Accurate Livestock Sciences, Rapperswil, Switzerland; Department of Animal Science, University of Pretoria, Pretoria, Gauteng, South Africa; Department of Animal Science, University of Pretoria, Pretoria, Gauteng, South Africa; Fonterra Research and Development Centre, Palmerston North, New Zealand; Sustainable Nutrition Initiative®, Riddet Institute, Massey University, Palmerston North, New Zealand; GOALSciences – Global Observatory on Accurate Livestock Sciences, Rapperswil, Switzerland; GOALSciences – Global Observatory on Accurate Livestock Sciences, Rapperswil, Switzerland; GOALSciences – Global Observatory on Accurate Livestock Sciences, Rapperswil, Switzerland; GOALSciences – Global Observatory on Accurate Livestock Sciences, Rapperswil, Switzerland; Department of Animal Science, University of Pretoria, Pretoria, Gauteng, South Africa; GOALSciences – Global Observatory on Accurate Livestock Sciences, Rapperswil, Switzerland

**Keywords:** interactive tool, livestock environmental impact, livestock production systems, livestock protein food basket

**Implications**

**The Animal Production System Evaluator tool for evaluating and comparing livestock production systems illustrates the contribution they can make to nationally adjusted protein food baskets in relation to different environmental impact indicators.** There is value in creating interactive tools for stakeholders, which facilitate informed decision-making in order to optimize productivity and sustainability in livestock production.
**The Animal Production System Evaluator tool builds on data sources from the Food and Agriculture Organization, the Global Livestock Environmental Assessment Model, and the PLANET Food System Explorer.**
**The tool aims to satisfy the following characteristics:**
*Multifaceted Assessment*: Evaluations of livestock production systems are required for integrating both nutritional contributions and environmental impacts to promote sustainable practices.
*Tailored Solutions*: Region-specific solutions that account for the diverse conditions across different areas are needed, moving away from a one-size-fits-all approach.
*Systemic Focus*: It is important to focus on entire production systems, rather than on animals, to ensure effective resource management and to mitigate environmental impacts.
*Global Relevance*: Livestock production’s dual role in providing nutrients and managing environmental impacts is essential for achieving sustainable development goals.

## Introduction

When evaluating livestock production contribution to society and its environmental impact, it becomes essential to consider production systems, rather than just focusing on animals. It is the system under which the animal operates that determines the kind of positive or negative influences it has on different environmental dimensions, and it is also the system that defines the effectiveness of converting low-value agricultural biomass into higher value food nutrients for a national food basket. The choice of systems can make more difference than the choice of species.

The distinction between livestock production systems is evident when comparing highly intensified industrial-scale systems with smaller-scale backyard systems or, in the case of ruminants, freely grazing animals with concentrated animal feeding operations. Industrial systems focus on high productivity and efficiency, relying on advanced technologies, precise management, and optimized resources to maximize output and minimize waste. In contrast, backyard systems emphasize sustainability, resilience, and local adaptability, often using low-cost technologies and community knowledge. These systems prioritize self-reliance and local breeds and contribute to food security and household income, especially in rural areas. For ruminants, when examining the Global Livestock Environmental Assessment Model (GLEAM) Dashboard version 3 (FAO, 2022b) for the world, it shows that a feedlot animal has an emission intensity of 60.35 kg CO_2_eq/kg protein, while a beef animal in a mixed system has an intensity of 361.04 kg CO_2_eq/kg protein, despite both being cattle.

Each system has different characteristics—such as animal weight, genetics, and performance—based on its unique resources and management practices. Industrial systems are designed for large-scale efficiency, while backyard systems integrate local resources and practices to ensure sustainable food production. The environmental and food security impacts of these systems vary not just because of the animals involved but the variety of inputs, highlighting the need for tailored evaluation.

To effectively assess and enhance the contributions of livestock to the global food supply and minimize their environmental impacts, it is essential to evaluate these systems comprehensively and consider their unique goals and characteristics. A one-size-fits-all approach would be ineffective, making it crucial to develop tailored strategies for each system.

The aim of this article and, more importantly, the interactive online tool it describes, is to review research on livestock production system classifications and highlight the importance of evaluating their contributions to national food baskets and environmental impacts. We introduce the tool to analyze and compare various livestock production systems on a regional basis. The tool aims to provide insights into the specific contributions of different livestock systems, enabling researchers and policymakers to optimize livestock production for enhanced food security and reduced environmental impacts.

## Existing Research on Defining Livestock Production Systems

Livestock production systems are defined and categorized by various international organizations, each contributing unique perspectives and methodologies. Key organizations in this field include the Food and Agriculture Organization (FAO), the Global Livestock Environmental Assessment Model (GLEAM), and the International Livestock Research Institute (ILRI).

- FAO categorizes livestock production systems based on geographical regions and agro-ecological zones, considering factors such as climate, resources, and management practices. These classifications, which include grassland-based, mixed farming and landless systems, provide a foundational understanding of global livestock practices and their regional variations (Robinson et al., 2011).
- GLEAM focuses on assessing the environmental impacts of livestock production systems worldwide. It integrates data on herd structures, feed intakes, manure management, land use, greenhouse gas (GHG) emissions, water use, and agro-ecological conditions to quantify the sustainability of different production systems. The model evaluates three production systems for cattle (grazing, mixed, and feedlot), two for other ruminant species (grazing and mixed), three for pigs (backyard, intermediate, and industrial), and three for chickens (backyard, layers, and broilers). GLEAM’s approach facilitates the comparison of ecological footprints across various livestock systems and regions (FAO, 2022a).
- ILRI conducts research on livestock systems, emphasizing their role in sustainable development and food security. Their classifications often incorporate socio-economic factors alongside environmental considerations, aiming to optimize livestock production for societal benefits while minimizing negative impacts (ILRI, 2024).

Other organizations, such as national agricultural research institutes and academic institutions specializing in livestock science, also contribute to defining and categorizing production systems. Their research often focuses on specific regional contexts or species-specific production practices.

## Introducing the Tool

**Figure 1.**
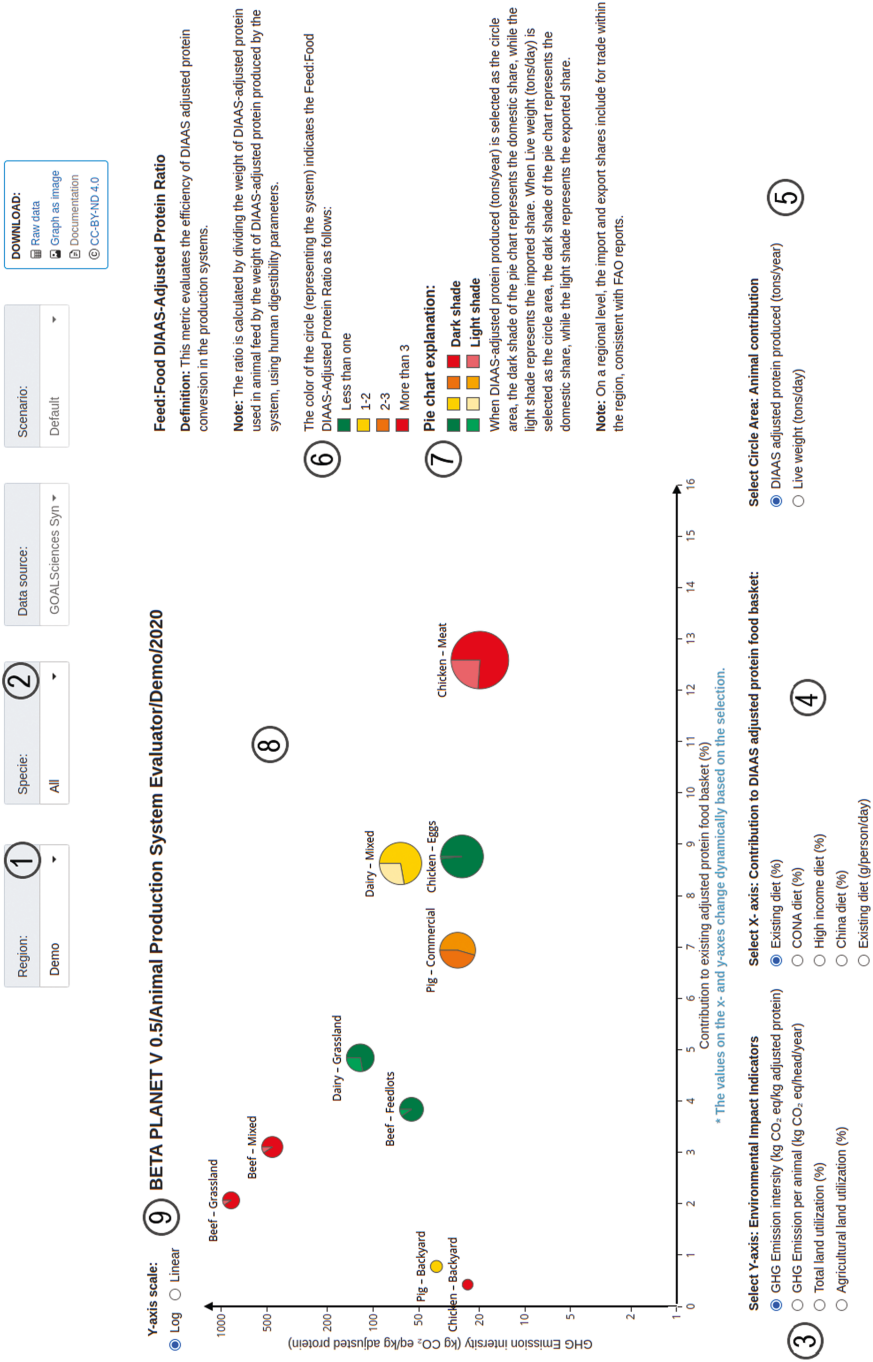
Example of the Animal Production System Evaluator (APSE) tool. 1.Region selector 2.Species selector 3.Environmental impact indicator selector 4.Food basket metrics selector 5.Circle area selector 6.Feed: Food DIAAS-Adjusted Protein Ratio 7.Pie chart explanation 8.Production system data points 9.Selector between logarithmic or linear view of y-axis.

The Animal Production System Evaluator (APSE) is an online interactive tool forming part of the PLANET Food System Explorer Platform available on goalsciences.org. The Beta-version 0.5 was released on 9 December 2024 and is freely accessible online. Figure 1 presents an example of the tool layout, axis, and visual data point representation. Updated releases will be provided continuously as more data become available and methodologies improve, with the aim of delivering data at a country level.

The APSE integrates nutritional metrics and environmental impacts with the aim of providing a multifaceted analysis of livestock production systems. Recognizing the diverse conditions across a regional basis, the tool emphasizes tailored solutions rather than a universal approach. The tool underscores the significance of production systems over individual animal assessments. By focusing on systems, it addresses the complexities of livestock production and promotes strategies that balance nutritional security and environmental sustainability. This integration facilitates informed decision-making by stakeholders across the agricultural sector.

## APSE Tool Interactivity

The APSE tool features selectors on both the x- and y- axes, allowing users to customize the variables displayed in the visualization. It also includes a circle area selector, accompanied by a pie chart that represents production share, offering a visual comparison of different systems. The color of each circle indicates the adjusted protein use efficiency of the production system relating to feed-food competition, providing an intuitive way to assess this important metric. These interactive elements work together to enhance the user’s ability to analyze and compare production systems.

### Environmental impact indicators: y-axis selector

Different livestock production systems exhibit varying degrees of environmental impact. For example, intensive systems may have lower GHG emissions but require more feed and water inputs per unit of animal, whereas extensive systems might impact biodiversity more as a result of land use expansion. As each country has its own resources, it is important to evaluate each separately for the environmental impacts.

In the APSE tool, the y-axis gives the option of four environmental impact indicator selectors, namely:

- Percentage of total land utilization;
- Percentage of agricultural land utilization;
- GHG emission intensity, kg CO_2_eq/kg DIAAS adjusted protein;
- GHG emission per animal, kg CO_2_eq/head/year.

### Land utilization

The land use data for each production system are sourced from the PLANET Food System Explorer platform base year 2020, which takes and processes its data from FAO “Land use” and “Crops and livestock products for area harvested” accounts. These data include the land required for the feed consumed by animals within each specific system, excluding any land used for imported feed. The information is then presented as a percentage of the total land or agricultural land, reflecting its utilization on a regional basis. See equations 1 and 2.

Equation 1:


$$\begin{array}{lll} Total\;land & \;utilization\;\left(production\;system\;x\right)\;\%\;\; & =\;\;\;\frac{Land\;used\;for\;feed\;\left(production\;system\;x\right)km^{2}}{Total\;available\;land\;\left(region\right)km^{2}}\times 100 \end{array}$$

Equation 2:


$$\begin{array}{lll} Agricultural & \;land\;utilization\;\left(production\;system\;x\right)\%\;\; & =\frac{Land\;used\;for\;feed\left(production\;system\;x\right)\;km^{2}}{Agricultural\;available\;land\;\left(region\right)\;km^{2}}\times 100 \end{array}$$

Tracking land use in livestock production is crucial for ensuring environmental sustainability, economic efficiency, and food security. It helps monitor and mitigate negative impacts like land degradation, biodiversity loss, and GHG emissions. Proper land use tracking also supports water resource management, optimizes agricultural productivity, and informs policy development. Overall, it ensures that livestock production meets current food demands without compromising future resources or the well-being of local communities (Phelps and Kaplan, 2017).

### Greenhouse gas emissions

GHG emission data are given on a regional basis per production system as calculated by the GLEAM dashboard version 3, base year 2015. Future releases of the APSE tool aim to give the data on a country-by-country basis, as they become available, provided by the scientific research community with consistency.

GHG emissions in livestock production systems are important because they significantly contribute to global warming, particularly through methane from enteric fermentation and nitrous oxide from manure management. These emissions intensify climate change, which can disrupt agriculture, water resources, and ecosystems. Reducing GHGs in livestock production systems is essential to minimize their environmental impact and promote more sustainable food production (FAO, 2023a).

### Water utilization

The first release of the APSE tool will not have water utilization available as a selector, but future releases aim to have blue water utilization per livestock production system available.

Water is a critical resource in livestock production systems, used for drinking, feed crops, and cleaning. Livestock farming can strain water resources through high consumption and pollution from runoff containing manure and chemicals. Proper water management is essential to ensure sustainability, protect water quality, and maintain the availability of clean water for both agricultural and human needs (Wisser et al., 2024). Eventually, we are aiming to also include an indicator for biodiversity.

The choice of these four dimensions of environmental impact—land use, GHG emissions, water utilization, and biodiversity—follows the main categories by which FAO tracks livestock impact, for instance in the 2023 FAO Report on Sustainable Livestock Transformation, page 18 (FAO, 2023b).

### Food basket contribution: x-axis selector

The concept of the food basket encompasses essential metrics crucial for assessing nutritional adequacy and health impacts. Key metrics include weight, energy content, protein content, and adjusted protein levels accounting for digestibility, amino acids, and essential micronutrients. These metrics collectively define the nutritional profile of food sources, including those derived from livestock production systems.

In nutritional science, identifying limiting nutrients—those critical for optimal health but often deficient in diets—is crucial. These nutrients, such as certain amino acids and micronutrients, play pivotal roles in metabolic processes and overall well-being. Adjusted protein, that considers digestibility and essential amino acid content, emerges as a vital measure for evaluating protein quality and nutritional value across different food sources. The importance of nutrient density is discussed by LeRoy et al. (2025) in this issue.

Livestock production contributes significantly to the food basket by providing high-quality protein, fatty acids, and micronutrients essential for human nutrition. The diversity in livestock products—meat, dairy, and eggs—offers varied nutritional benefits that complement plant-based diets, addressing nutritional deficiencies and enhancing dietary diversity. The primary role of livestock-derived products is to serve as a key source of essential nutrients in the national food basket. That is the reason why we chose Digestible Indispensable Amino Acid Score (DIAAS)-adjusted protein, which evaluates the digestibility and availability of individual essential amino acids in food, as the proxy variable for the contribution to the nutrient density of a national food basket.

The corresponding data are generated by the PLANET Food System Explorer (2024) platform, whose primary sources of empirical data are Food Balance sheets and Supply Utilization Accounts from FAO, base year 2020. The livestock production systems’ food contributions are calculated and then divided by the total food basket to determine the percentage, as outlined in equation 3.

Equation 3:


$$\begin{array}{lll} Contribution & \;to\;existing\;adjusted\;protein\;food\;basket\left(production\;system\;x\right)\%\; & =\;\;\;\frac{Adjusted\;protein\;\left(production\;system\;x\right)\;gram\;per\;capita\;per\;day}{Total\;adjusted\;protein\left(region\right)gram\;per\;capita\;per\;day}\times 100 \end{array}$$

Different food baskets are provided to choose from, reflecting the various options for defining what the food basket should include. The APSE tool offers the following selectors on the x-axis for food basket metrics:

- Contribution to existing DIAAS-adjusted protein food basket (%);
- Contribution to required DIAAS-adjusted protein food basket (%) – as defined by CONA diet;
- Contribution to required DIAAS-adjusted protein food basket (%) – as defined by high-income countries diet;
- Contribution to required DIAAS-adjusted protein food basket (%) – as defined by the Chinese diet;
- Absolute contribution to DIAAS-adjusted protein food basket (g/person/day).

(The high-income countries and Chinese diets are derived from FAOStat data. These fully represent adequate diets with varying compositions, while the CONA diet, sourced from Bai et al. (2022), represents a least-cost diet that meets dietary reference intake requirements.)

### Contribution: circle area selector

When comparing two or more livestock production systems, the units of animal live weight (tons per day) or adjusted protein production (tons per year) can be selected. The contribution of the systems relative to each other is visualized by the circle area. The data are generated by the PLANET food system explorer platform on a regional basis, primarily on the basis of FAOstat and GLEAM data. The animal weight option makes the circle size comparison relevant to the y-axis of environmental impact. The protein production option makes it relevant to the x-axis of nutritional contribution.

Within the circle, a pie chart is displayed comparing the contribution of export and domestic production for the system with reference to live weight or the contribution of import and domestic production with reference to adjusted protein produced.

### Feed-food competition: circle color

The circle color indicates the adjusted protein use efficiency of the system. This is calculated by dividing the amount of adjusted feed protein consumed by the amount of food protein produced by the livestock production system in terms of human suitable adjusted protein. The Feed-Food Competition Circle uses a color legend to represent different ratios: green indicates a ratio of less than 1, yellow represents a ratio between 1 and 2, orange signifies a ratio between 2 and 3, and red indicates a ratio greater than 3. This legend helps interpret the degree of competition between feed used for animal production and food available for human consumption.

Feed-food competition is important because it highlights the tension between using land and resources to grow crops for livestock feed vs. crops directly for human consumption. As global food demand rises, prioritizing feed can reduce the availability of arable land for food crops, potentially leading to higher food prices and variability in food security. Addressing this competition is crucial for ensuring that agricultural systems are both sustainable and capable of meeting the nutritional needs of a growing population (Breewood and Garnett, 2020).

## Outlook and Conclusion

Understanding and evaluating livestock production systems through integrated tools, such as the APSE tool, and metrics is essential for addressing global challenges related to food security, nutrition, and environmental sustainability. By focusing on production systems rather than animals, stakeholders can develop tailored strategies that optimize nutritional contributions while mitigating environmental impacts. This approach supports sustainable development goals and promotes resilience in agriculture, recognizing the diverse conditions and needs across different regions worldwide.

**Figure 2.**
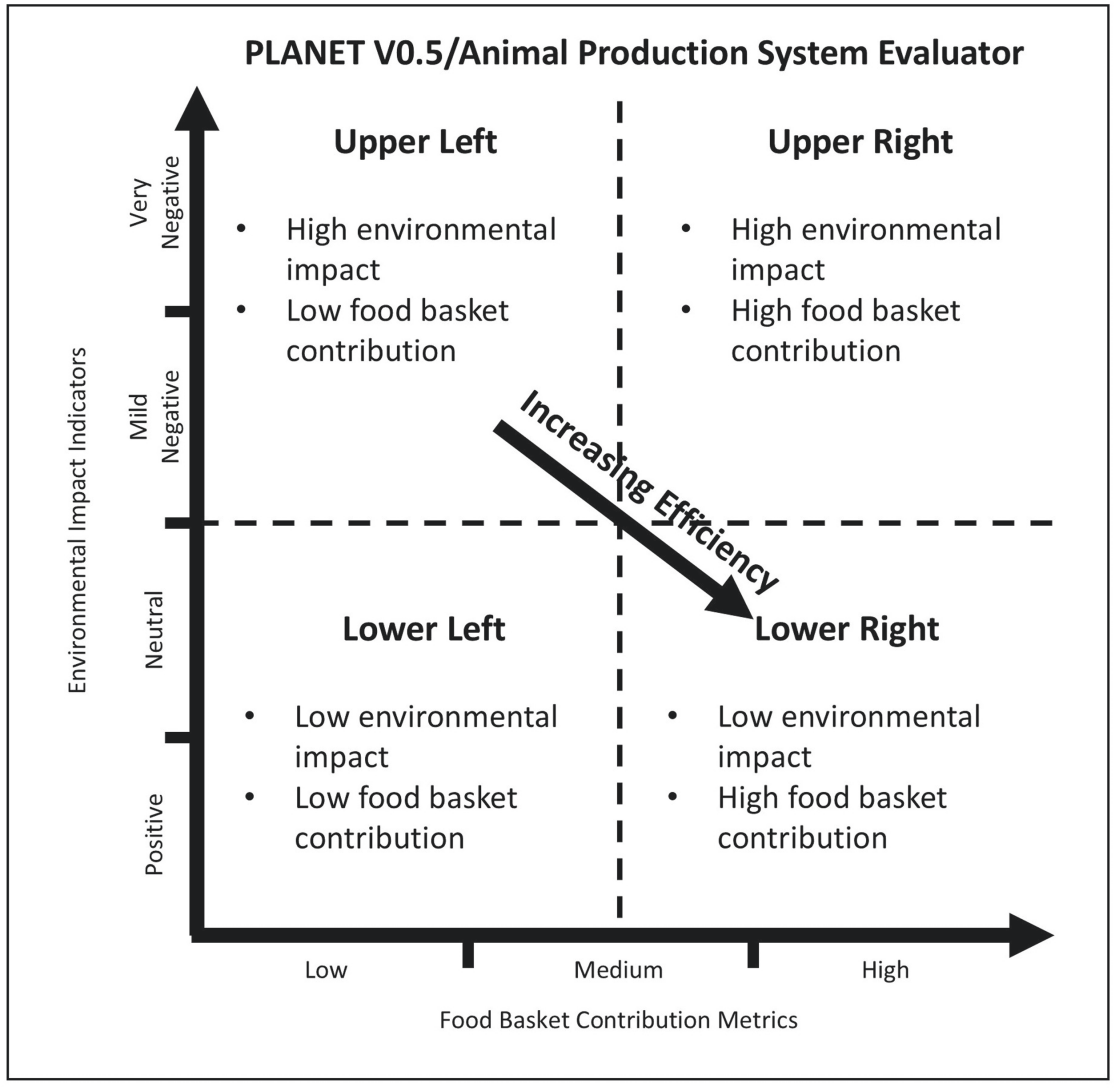
Interpretation of four quadrants in APSE tool.

The ultimate aim of the APSE interactive tool is to illustrate and interrelate the multiple dimensions by which livestock production systems are relevant to a society. We hope this tool will be able to assist public and private decision-makers to optimize their national livestock strategies. Generically, the implication is that if the overall system is more resource-efficient, the more production systems can be moved from the upper-left corner of the matrix to the lower-right corner (Figure 2).

More details on the methodology and a case study can be found in the Supplemental information. It is important to note that the effectiveness of the APSE tool is dependent on the quality of the available data. The primary limitation of the tool is the accuracy of this information. As more accurate data become available, the tool will be updated to improve its precision. Additionally, some data are not yet available in several countries. We welcome any contributions from the global scientific community to contribute data to making the tool more comprehensive.

## Supplementary Data

Supplementary data are available at *Animal Frontiers* online.

vfaf008_suppl_Supplementary_Material

## About the Author(s)

**Elna de Lange** is a researcher at GOALSciences, with a passion for animal nutrition and a background in animal science and feed manufacturing. With a BSc (Agric) in Animal Science from the University of Pretoria, she began her career as an animal nutritionist in the feed manufacturing industry, where she gained a thorough perspective of the South African animal feed industry. Elna combines her expertise in animal nutrition and her experience in data analysis to bring relevant, accurate, and objective data to the GOAL Sciences. In her personal time, she focuses on improving her horsemanship and living life authentically. Corresponding author: elna.delange@goalsciences.org

**Bohdana Kalinovska** earned her Master’s degree in Computer Science from the National Technical University of Ukraine «Igor Sikorsky Kiev Polytechnic Institute» in Kyiv. Her passion for data processing, regression analysis, data forecasting, and data visualization has helped her gain a wealth of knowledge and expertise in the field. Bohdana has a strong academic background in programming languages, data structures, algorithms, and software development principles, which enables her to effectively solve complex tasks in the industry.

**Taras Iliushyk** received his Master’’ degree in computer science, specializing in computer technology in Biology and Medicine at the National Technical University of Ukraine «Igor Sikorsky Kiev Polytechnic Institute». Since then, he has deepened his knowledge of coding in different software languages, with a special emphasis on creating data science applications for the processing of big data sets.

**Enrike Maree** is a Researcher and Communications Manager at GOALSciences. She holds a BScAgric in Animal Science from the University of Pretoria and has submitted her thesis for an MScAgric in Animal Science, focusing on sustainability indices for the agricultural industry at the same institution. Her previous experience includes roles as a nutrition intern and a technical advisor at a leading feed company. In her current role, she conducts research aimed at enhancing sustainability in livestock production and the broader agricultural sector. Additionally, she engages in consumer education through AgreeToThisAgri. Her research interests encompass livestock sustainability, food security, sustainable environments, and the socio-economic impacts of sustainable agriculture, with a vision of promoting a holistic, sustainable food system.

**Naomi Lupton** is a graduate of the University of Pretoria in South Africa, having completed both a bachelor’s degree in Animal Science as well as a master’s degree in Production Physiology with a focus on Meat Science and Animal Welfare. During her studies, she aided the Department of Animal Science and Future Africa Experimental Farm as a tutor, research assistant, and photographer. She is currently employed as a Researcher at the Global Observatory for Accurate Livestock (GOALSciences) and is interested in topics relating to animal and human nutrition, animal welfare, and sustainable agriculture.

**Andrew Fletcher** is an Honorary Fellow at the Riddet Institute, where he is one of the founders of the Sustainable Nutrition Initiative (SNi). SNi aims to provide robust scientific evidence for the sustainable food systems debate. He has had a long career in Fonterra—in a variety of research management and strategy roles within the cooperative and across the wider New Zealand dairy industry—and is currently Program Leader Sustainable Food Systems, which includes work on on-farm GHG mitigation technologies and how changes in on-farm technology are captured in product footprints. Within SNi, his focus is on the development of tools and models—such as the DELTA Model—that enable users to develop their understanding of the global food system and the contribution of foods and food production technologies to sustainable nutrition.

**Peer Ederer** is the founder and director of GOALSciences, the Global Observatory of Accurate Livestock Sciences, which has the mission to research and communicate scientific evidence about the role of animals in the global food system. He has an MBA degree from Harvard University, a PhD in financial economics, and holds an adjunct professorship for innovation studies. He has extensive experience in providing strategic advice to private companies and public bodies in the global food system and is a frequent presenter on related topics. He has been engaged in scientific research in cooperation with leading universities from around the world and is a member of the Scientific Council of the World Farmers’ Organisation.

**Lindeque du Toit** is a Senior Lecturer in the Department of Animal Science at the Faculty of Natural and Agricultural Sciences, University of Pretoria. He holds a BSc (Agric) in Animal and Pasture Science, an MSc (Agric) in Animal Nutrition, and a PhD in Animal Nutrition all from the University of Pretoria. His research area is focused on meeting the challenge to increase the production of quality livestock protein and fiber to meet increasing demand, adapt to environmental changes, and reduce the impact of livestock production systems on the environment. This includes developing greenhouse gas emission factors for various classes of livestock in South Africa and exploring practical mitigation strategies. He teaches courses in livestock production systems, pasture and range management, and animal nutrition. He is currently a serving member of the South African Society for Animal Science and a member of the South African Council for Natural Scientific Professionals.
