# Supplementary material for: Shifting the focus from animal species to livestock production systems: an interactive tool for evaluating food contributions relative to environmental impacts
Source: Anim Front. 2025 Apr 5;15(1):72–9. doi: 10.1093/af/vfaf008 (PMC11971519; doi:10.1093/af/vfaf008)
Supplement: vfaf008_suppl_Supplementary_Material [file vfaf008_suppl_supplementary_material.pdf]

Supplement to Article:

Shifting the Focus from Animal Species to Livestock Production Systems: An Interactive Tool for Evaluating Food Contributions Relative to Environmental Impacts

In Animal Frontiers 2025, Issue #1

Elna de Lange, Lindeque du Toit, Andrew Fletcher, Taras Iliushyk, Bohdana Kalinovska, Naomi Lupton, Enrike Maree and Peer Ederer

Online interactive access to the Animal Production System Evaluator (APSE):
https://goalsciences.org/food-system-explorer/animal-production-system-evaluator

APSE Tool Example

To assist with the interpretation of the APSE tool, refer to Figure 1, which provides an overview of the four quadrants within the tool.

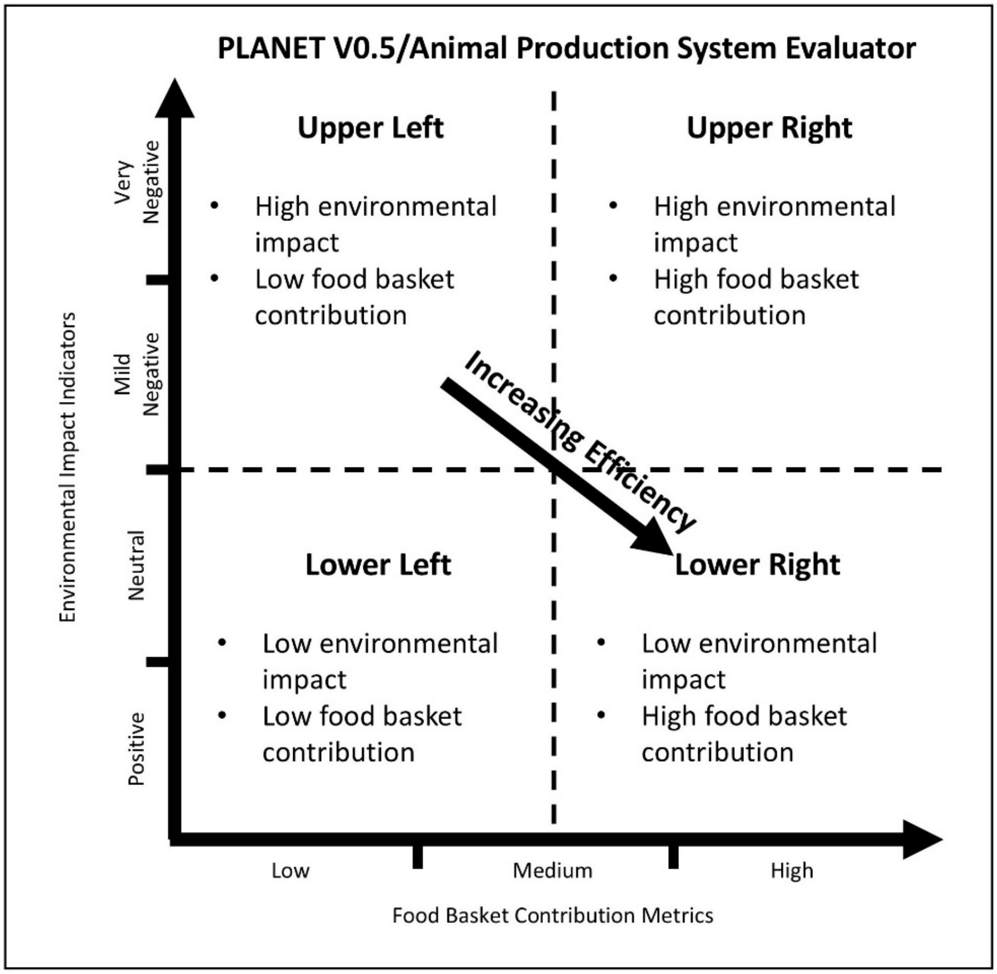

Figure 1: Interpretation of four quadrants in APSE tool.

We will now proceed to work through Figure 2 as an example of how to interpret the tool's results. Table 1 provides an overview by comparing the four livestock systems (A, B, C and D) as shown in Figure 2.

Table 1: Overview interpretation of Figure 2 livestock production systems

System	Environmental impact	Food contribution	Feed: Food competition	Pie chart (adjusted protein produced)
A	High	Low	Low	25% import share
B	High	High	High	20% import share
C	Low	Low	Medium	None
D	Low	High	Medium	50% import share

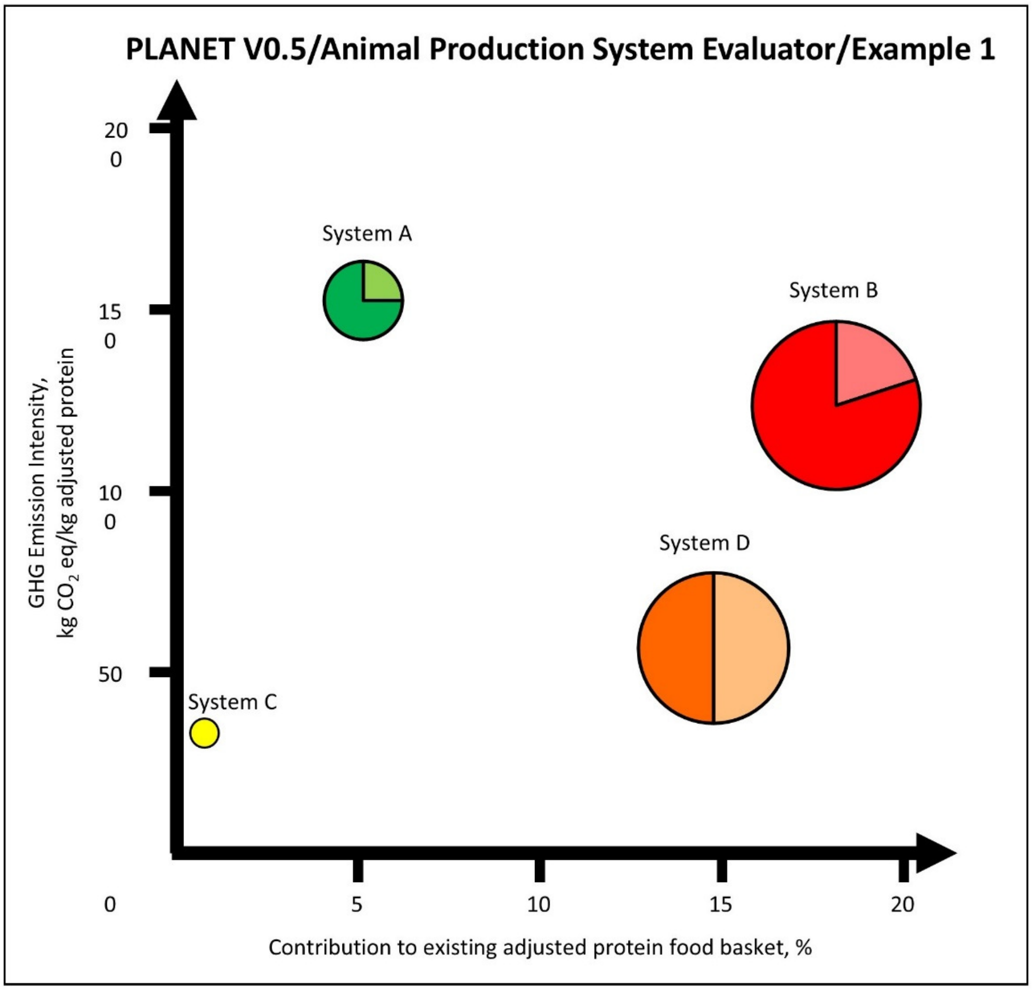

Figure 2: APSE tool example comparing four livestock production systems.

Analysis of Systems:

System A has the highest environmental impact but contributes relatively little to food production, with low feed-food competition and a moderate reliance on imported production (25%). Strategic Recommendation: Improvement efforts should focus on reducing its environmental footprint without significantly altering its role in the food system.

System B is crucial for improvement, exhibiting a high contribution to food production (adjusted protein) alongside a significant environmental impact. It also faces considerable feed-food competition, indicating that changes here could yield substantial benefits in both reducing environmental impact and optimising food production. Its relatively low reliance on imports (20%) makes it a prime candidate for targeted interventions. Strategic Recommendation: Prioritise efforts to reduce its environmental footprint while enhancing feed efficiency and minimising feed-food competition.

System C shows a low environmental impact and low contribution to food production, with a medium level of feed-food competition and no reliance on imports. While it is not a major environmental concern, it does not significantly enhance food security. Strategic Recommendation: Optimisation efforts should aim to increase food production efficiency while maintaining its low environmental footprint.

System D balances low environmental impact with high food contribution but has the highest reliance on imported production (50%), which poses a threat regarding food security and sustainability. Strategic Recommendation: Focus on reducing import dependency while sustaining or enhancing food contribution, leveraging its strengths in low environmental impact.

The analysis of Figure 2 using the APSE tool underscores the importance of targeted interventions in livestock production systems. By focusing on System B for significant improvements and considering strategic enhancements in Systems D, A, and C, the overall sustainability and efficiency of the food system can be greatly improved.

Livestock Production systems in APSE Tool

The APSE tool integrates livestock production systems from the GLEAM model. For pigs, the systems include backyard and commercial (a combination of industrial and intermediate) operations. For chickens, the categories are backyard, broiler, and layer systems. The cattle systems are classified into beef (feedlots, grassland and mixed) and dairy (grassland and mixed).

Overview of Factors Used in Defining Livestock Production Systems

Livestock production systems are defined by a combination of factors that encompass biological, economic, and environmental considerations. Understanding these factors is crucial for categorising and optimising livestock management practices. Table 2 gives an overview of the factors that the APSE tool aims to incorporate in its measurements. Understanding and integrating these factors into livestock production systems allows for tailored approaches that optimise productivity, economic efficiency and sustainability while meeting market demands and ensuring animal welfare.

Table 2 : Overview of factors used in comparing livestock production systems

Factors	Detail
**Species**	The APSE tool includes cattle, chickens and pigs. Different species have distinct physiological characteristics, nutritional needs and productivity potential that influence management strategies and resource allocation.
**Herd Structure**	The APSE tool aims to provide a foundational herd structure for each production system. The organisation and composition of animal groups within a herd or flock have an impact on breeding programmes, disease control, feeding strategies and labour requirements, while also influencing overall efficiency and management decisions.
**Resources**	The availability and quality of resources such as land, water, feed and veterinary services significantly influence the type and intensity of livestock production systems. The APSE tool incorporates land and feed use into its analysis.
**Product (Meat/Milk/ Eggs)**	Primary outputs dictate breeding goals, nutritional management and processing methods. Different products require specific production systems tailored to optimise both quality and quantity. This is considered in the APSE tool.
**Management Practices**	Techniques and strategies in livestock husbandry, such as housing systems, feeding regimes, disease prevention and waste management, directly impact animal welfare, productivity and environmental sustainability. The APSE tool includes considerations for housing and feeding.
